# Characteristics and Mitigation Measures of *Candida* *auris* Infection: Descriptive Analysis from a Quaternary Care Hospital in Saudi Arabia, 2021–2022

**DOI:** 10.1007/s44197-023-00154-9

**Published:** 2023-10-23

**Authors:** Hala A. Amer, Sarah AlFaraj, Kholoud Alboqami, Faleh Alshakarh, Mona Alsalam, Deva Kumar, Juhaina Altayieb, Antisar Alsunid, Nazia Khanum, Nadeem Gul Dar, Muhammad Badawi, Hassan Abdallah, Ziad A. Memish

**Affiliations:** 1 Prevention and Control of Infection Administration, King Saud Medical City, Riyadh, Saudi Arabia; 2 Community Medicine Research Department, National Research Center, Cairo, Egypt; 3 Research and Innovation Center, King Saud Medical City, Riyadh, Saudi Arabia; 4 College of Medicine, AlFaisal University, Riyadh, Saudi Arabia; 5 Hubert Department of Global Health, Rollins School of Public Health, Emory, University, Atlanta, USA; 6 Division of Infectious Diseases, Kyung Hee University, Seoul, Korea

**Keywords:** *C*. *auris*, Risk factors, Preventive measures

## Abstract

**Objective:**

To analyze the characteristics of *C*. *auris* cases, and to describe the interventions applied for improving the diagnosis and controlling the transmission.

**Method:**

Medical records of *C*. *auris* cases reported between January 2021 until June 2022 at King Saud Medical City (KSMC), Riyadh, Kingdom of Saudi Arabia have been reviewed. We analyzed the demographic and clinical characteristics of the cases to illustrate the possible contributing factors with *C*. *auris* infection. A multidisciplinary committee has been formulated to investigate the potential source of the outbreak among clusters of cases in the intensive care units (ICU). A bundle of mitigation measures has been applied which was successful to contain the outbreak.

**Results:**

During the study period, a total of 129 cases of *C*. *auris* were identified, their mean age is 47 ± 22.3 SD, and 72.1% are males. 57% of cases were colonized, all of them were identified through active screening. A number of comorbidities were present including 27.9% were having hypertension, 27.1% with diabetes, 22.5% with COVID-19 and 20.2% with respiratory diseases. The average length of stay before reported positive was 36.23 days. 78.3% of those patients were in the critical care unit, 73.6% with vascular catheter, 88% with urinary catheters and 66.7% with mechanical ventilation. The vast majority of patients were using multiple antibiotics (86%). As per the univariate logistic model, risk factors significantly associated with mortality were (Age, Trauma RTA, ICU, Vascular Access, Foley Catheters, Mechanical Ventilation, Tracheostomy and Endotracheal Tubes) with *p* values (0.0038, 0.0159, 0.0108, 0.0122, 0.0071, &lt;.0001, 0.0148 and 0.0107), respectively. Multivariate logistic regression showed that having a Foley Catheter was the only statistically significant factor associated with mortality.

**Conclusion:**

This retrospective analysis  highlights the main characteristics associated with *C*. *auris*-infected patients. In addition, it highlights the effectiveness of the bundle of mitigation strategies applied to limit the spread of *C*. *auris* in healthcare facilities.

## Introduction

*Candida auris* is a multidrug-resistant fungal pathogen although only described in 2009 in Japan, now has a near-global distribution. It was added in late 2022 to the World Health Organization (WHO) first published fungal priority pathogens list, and classified as an urgent public health threat by the Centers for Disease Control and Prevention (CDC) in 2021 [1–3]. Until December 31, 2020, *C. auris* infections have been reported in 44 countries globally [4]. First identified in Kingdom of Saudi Arabia (KSA) in 2018 and was made nationally notifiable in 2021 [5].

Using the biochemical methods such as Analytical Profile Index (API) strips or VITEK-2 system, usually misidentifies *C*. *auris* as other Candida species or Saccharomyces. Although the development of matrix-assisted laser desorption/ionization time-of-flight mass spectrometry (MALDI-TOF MS) and sequencing strategies have helped in the rapid and accurate diagnosis of *C. auris*, most parts the world does not have the infrastructure to carry out these techniques [6, 7]. In addition, it is likely that there is a large volume of unpublished data pertaining to *C. auris* infections, and the number of infected patients may be far higher than that reported in the literature. Therefore, this is considered by many as an “invisible pandemic” [8].

Some isolates resistant to all three major antifungal classes, and the CDC has reported two independent outbreaks of pan-resistant C. auris strains [2].

Since *C. auris* has a high mortality rate, ambiguous mechanisms of spread, frequent misidentifications since conventional methods do not detect it, and multidrug resistances, therefore, it can now be considered a major global public health threat [9]*.* The worldwide epidemic of *Candida auris* infections was exacerbated by the ongoing pandemic of coronavirus disease 2019 (COVID‐19) [10].

Our study aim is to analyze cases of *C. auris* reported between 2021 and 2022 at King Saud medical City (KSMC) and to describe the interventions applied to improve diagnostics and prevent and control the transmission.

## Methods

Descriptive analysis and outbreak investigation carried out at KSMC, one of the main Ministry of Health healthcare facilities in the central zone of Saudi Arabia.

It has a total capacity of around 1400 beds with 120 Intensive Care Unit (ICU) beds, and it serves as a trauma center and a referral hospital for the majority of KSA geographical zones.

Medical records of all cases reported as *C. auris* between January 2021 and June 2022 were reviewed. Patient demographic data, underlying diseases, risk factors for infection, mortality were been analyzed.

The subjects were anonymously coded by serial numbers for data analysis. The participants’ names and medical record numbers were kept confidential and not accessible except to principal investigators.

Interventions and protocols applied to control the spread of the emerging *Candida auris* infections included: formulation of a multidisciplinary committee headed by the hospital CEO and involved infection control, infectious diseases, intensive care, nursing, environmental services, pharmacy, and laboratory services to investigate the potential source of the outbreaks among clusters of cases in the intensive care units (ICU). A bundle of containment measures were applied at ICU level including, *C. auris* admission screening, point prevalence testing every two weeks, patients and staff post-exposure screening, environmental sampling, high level disinfection protocol, dedication of patients’ placement and equipment of care, enhancing patient hygiene, universal contact precautions, and frequent education and auditing of infection control practices. Additionally, introduction of advanced identification technology (MALDI-TOF MS) was one of the main recommendations raised by the committee which was available by end of 2022.

All the statistical analysis was done by the software SPSS “IBM SPSS Statistics for Windows, version 23 (IBM Corp., Armonk, N.Y., USA)”. Frequency analysis performed for all variables.

The Chi square test was used for univariate analysis to identify indicators that had a significant association with the cases’ outcome. Indicators with *P* values less than 0.05 were included in a multivariate regression model.

The study has been conducted in compliance with KSMC institutional Research Board regulation and Waiver of consent has been granted.

## Results

A total of 129 cases of *C. auris* were identified between January 2021 and June 2022. The mean age of the subjects included in this study is 47 ± 22.3 SD (range 1–88), and Males represent 72.1%. The average length of stay before reported positive culture was 36.23 days (Std 48.13, range 1–365). Among this sample of 129 patients with *Candida auris*-positive specimen, 57% were considered colonized identified through active surveillance screening either upon admission to ICU or post-exposure to a positive *C. auris* case, sites colonized included axilla/groin in 35.7% % of cases and nasal in 27% of cases. Out of the specimens tested for antifungal resistance, 10% were resistant to for Fluconazole, 2% for Amp-B and 1% for Caspofungin (Table 1).

**Table 1:** Demographics and microbiological profile

	Count (%)
Age	Mean = 47 year
	SD = 22.3
Gender	
Female	36 (27.9)
Male	93 (72.1)
Axilla-Groin	46 (35.7)
Nasal	36 (27.9)
Rectal	11 (8.5)
Blood	18 (14)
Urine	21 (16.3)
Wound/other	26 (20.2)
Sputum	4 (3.1)
Colonized/infection	74 (57.0) 55 (43.0)
Fluconazole resistant	13 (10.0)
Amp-B resistant	2 (2.0)
Caspofungin resistant	1 (1.0)

In this sample of patients, a number of comorbidities were present including 27.9% with hypertension, 27.1% with diabetes, 22.5% with COVID-19 and 20.2% with respiratory diseases other than COVID-19 (Table 2). As is shown in the risk factors analysis, 78.3% of patients were in ICU, 73.6% with vascular catheter, 88% with urinary catheter and 66.7% with mechanical ventilation. The vast majority of patients were using multiple antibiotics (86%) (Table 3).

**Table 2:** Underlying Diseases

	Count (%)
COVID-19	29 (22.5)
Diabetes Mellitus	35 (27.1)
Hypertension	36 (27.9)
Respiratory Diseases	26 (20.2)
Cardiovascular Diseases	11 (8.5)
Neurological Disorders	26 (20.2)
Kidney Diseases	15 (11.6)
Gastrointestinal Tract Diseases	15 (11.6)
Oncology	5 (3.9)
Trauma/ RTA	27 (20.9)
Burn	5 (3.9)

**Table 3:** Risk factors

	Count (%)
ICU	101 (78.3)
Vascular catheter	95 (73.6)
Urinary catheter	110 (88.0)
Mechanical ventilation	82 (66.7)
Endotracheal Tube	44 (34.1)
Tracheostomy	48 (39.0)
Receiving multiple antibiotics	111 (86.0)
Total Parenteral Nutrition	26 (21.0)

Each one of the factors was entered in a separate univariate logistic model to assess its effect on mortality. Old age, critical care cases, admission diagnosis as road traffic accident (RTA), having a medical device including vascular access, foley catheters, mechanical ventilation, tracheostomy and endotracheal tubes were) were identified to significantly increasing the risk of mortality among *C*. *auris* cases with *p* values (0.0038, 0.0159, 0.0108, 0.0122, 0.0071, 0.0001, 0.0148 and 0.0107), respectively. Then, these statistically significant risk factors were all entered in one multivariate logistic regression where only (Foley Catheters) was statistically significant with *p* value (0.0168) (Table4).

**Table4:** Factors that may be associated with patient mortality:

Factor	Univariate	Multivariate
	*P* value	*P* value
Gender	0.5196	
COVID-19	0.5488	
Age	0.0038	0.1188
Diabetes mellitus	0.2638	
Hypertension	0.1837	
Respiratory diseases	0.2323	
Cardiovascular diseases	0.1679	
Neurological disorders	0.0626	
Kidney disease	0.0616	
Gastrointestinal tract disease	0.7769	
Trauma/RTA	0.0159	0.0777
Axilla groin	0.1428	
Nasal specimen	0.1399	
Blood specimen	0.4898	
Urine specimen	0.1659	
ICU	0.0108	0.7519
Vascular access	0.0122	0.5107
Foley catheters	0.0071	0.0168
Mechanical ventilation	< 0.0001	0.0825
Chest tube	0.6986	
Tracheostomy	0.0148	0.0710
Endotracheal tubes	0.0107	0.1735
Antibiotic	0.9239	
TPN	0.4492	
IV medications	0.6111	
Infected	0.0670	
Colonized	0.0670	

Hundreds of exposed staff were screened for *C. auris*, none of them turned positive. Environmental sampling for *C. auris* revealed positive results from glucometer and body thermometer used between patients. The trend of *C. auris* cases gradually decreased following the strict implementation of the mitigation measures.

## Discussion

This descriptive study investigated the cases of *C. auris* reported at KSMC between 2021 and 2022 which is one of the main healthcare facilities in Riyadh region with the largest ICU, and having an access for the laboratory resources for *C. auris* identifications. Additionally, the active surveillance for *C. auris* for all ICU admissions which was launched in Sept, 2021 had a significant role in increasing the identification of cases.

Among this study subjects, almost three fourth were males and the age of our cases ranged between 1 and 88 years with 6% being pediatrics (below 14 Ys) and 30% were considered elderly (above 60 Ys). Similarly, predominance of male patients (16 out of 17) reported by Asadzadeh et al., in their study of characteristics of *Candida auris* isolates from immunocompromised patients in a hospital in Kuwait and (41.1%) patients were > 70 years of age [11].

More than half of our study subjects (57%) were considered colonized cases detected from active screening, majority of them were axilla and groin colonization followed by nasal colonization (35.7% vs 27.9%, respectively). Some of this screening was performed as part of routine ICU admission screening started in September 2021 where 14 colonized cases were reported from 1864 admissions (0.75%) during our study period. Other indications for active screening were either following exposure to a positive *C. auris* case or upon physician request for septic screening. Within our study sample, three clusters of cases defined as outbreaks were reported in the ICU. The delay in detection, the prolonged turnaround time for the *C. auris* culture result, the inaccuracy in identifying candida species as well as some gaps in infection prevention practices—mainly the ineffective cleaning of surfaces and equipment—enhanced the transmission among those reported clusters.

Colonization with *C. auris* has been studied by *Karen S. *et al. who reported that between August 26, 2016 and November 7, 2017, 114 individuals colonized/screened with *C. auris* were identified (81% with axilla/ groin and 64% with nasal colonization; 60% had both sample sites positive within 7 days). These cases comprised 7% of the 1668 total individuals screened during 72 PPS and contact tracing efforts at 41 Healthcare Facilities [12].

22.5% of our cases were diagnosed with confirmed SARS-CoV2 infection which required ICU admission*.* Some studies have investigated the rise of fungal infections, especially the multidrug-resistant types/species in the era COVID-19. In their review about *C. auris* among critically ill COVID-19 cases in India, Chowdhary A et al. elaborated that candidemia in those kind of patients where mostly due to *C. auris* rather than *C. albicans* [13].

An increase of *C. auris* screening and clinical cases during the COVID-19 pandemic has been reported by California Department of Public Health in southern California region [14].

This rise in the *C. auris* cases has been justified by the resources constrains—such as staffing shortages and insufficient supply of personal protective equipment—during the pandemic that affect the adherence to essential infection control practices. Inadequate hand hygiene, contamination of the surrounding environment, use of shared medical equipment, and extended use of gowns and gloves are some of the gaps in infection prevention measures that likely contributed to *C. auris* transmission [15, 16].

The most common comorbidity reported among our study subjects are DM and hypertension (around 27% each) followed by neurological condition and history of trauma (around 20% each), then Kidney and GIT disease each represented in around 10% of our cases.

DM and hypertension are prevalent in Saudi community. Considering the complications of those chronic diseases that may require ICU admission and the depressive effect of DM on the immune system, those candidates are mostly immunocompromised and vulnerable for fungal infections such as *C. auris*.

The percentage of neurological condition and history of trauma among our study subjects is representative of the type of patients admitted in our ICU as our institution serves as trauma center and has specialized neuro critical care service/section. Additionally, those categories of patients went through multiple surgical procedures that exposed them to the risk of HAIs.

Among the *C. auris* patient population studied by Alicic et al., 18.4% had kidney disease, which was higher than the percentage reported in our study. They have also found that kidney disease is an important risk factor that affects mortality in *C. auris* infection patients. The association of kidney disease with mortality in *C. auris* cases may be justified by the fact that patients with kidney disease often suffers from diabetes and most of them have low protein and malnutrition, which leads to a decline in immunity, in addition to the frequent use of immunosuppressant in the treatment process of patients with kidney disease [17, 18].

Receiving antibiotics within 14 days prior to *C. auris*-positive specimen, ICU hospitalization, and using medical devices including central venous catheter, urinary catheter and mechanical ventilation are multiple risk factors for infection have been reported in high percentage among our study subjects. These factors represent high risk factors for *C. auris* infection and are consistent with the risk factors espoused by global experts in relation to *C. auris* emergence and transmission [19].

The mortality rate among our study subjects was 43.4% occurring within 20.13 days (std 16.45, range 1–71) of the date the positive *C. auris* specimen.

Patients on invasive devices such as mechanical ventilation, central vascular line and urinary catheter are at higher risk of acquiring Candida spp. infections as reported in many previous studies [20].

In our current report, it was found that invasive devices are significantly associated with higher mortality among *C. auris* patients in addition to other factors such as older age and ICU admission. However, as per the multivariate logistic regression, having Foley Catheters was the only factor which remains statistically significant with mortality (*p* value: 0.0168).

There are some limitations pertaining to this study. One of the main limitations is the non-availability of genotyping testing to investigate the epidemiological link between the reported cases. Other limitations include incomplete data about antifungal sensitivity pattern and lack of information about some *C. auris* risk factors reported in other studies such as history of abdominal surgical procedure.

Our findings have significant implications infection prevention and control measures in healthcare settings. *C*. *auris* was found to be an opportunistic infection for hospitalized patients on invasive devices in critical care units. Minimizing the risk of acquiring *C*. *auris* infections among such vulnerable group requiring strict adherence to essential infection prevention measures including hand hygiene, proper use of PPE and limiting the insertion and the duration of invasive devices use to the minimum with proper care of those devises. Considering high level disinfection for environment and equipment is also crucial.

In summary, this retrospective analysis elaborates the main characteristics associated with *C. auris*-infected patients. In addition, it highlights the effectiveness of the bundle of mitigation strategies applied to limit the spread of *C. auris* in healthcare facilities.

## Data Availability

Data will be available on a reasonable request.

## Abbreviations

*C*. *auris*: *Candida* *auris*
COVID-19: Corona virus disease 2019
KSMC: King Saud Medical City
MOH: Ministry of Health
ICU: Intensive care units
RTA: Road traffic accident
API: Analytical Profile Index
MALDI-TOF MS: Matrix-assisted laser desorption ionization time-of-flight mass spectrometry
